# From Confidence to Commitment: Self-Perceived Competence and the Motivational Pathways to Intellectual Engagement with AI Learning Tools

**DOI:** 10.3390/jintelligence14090220

**Published:** 2026-09-14

**Authors:** Fahad Alsudairi, Arwa Mohammed Asiri, Mohammed Khojah, Sabah Abdullah Al-Somali, Dana Bakry, Khalid Alqarni, Mohammed Alsaigh

**Affiliations:** Department of Management Information Systems, King Abdulaziz University, Jeddah 21589, Saudi Arabia; amaasiri@kau.edu.sa (A.M.A.); mmkhojah@kau.edu.sa (M.K.); saalsomali@kau.edu.sa (S.A.A.-S.); dbakry@kau.edu.sa (D.B.); khalqarni@kau.edu.sa (K.A.); oalsaigh@kau.edu.sa (M.A.)

**Keywords:** self-efficacy, technology acceptance, intellectual investment, motivation, enjoyment, artificial intelligence, higher education

## Abstract

Artificial intelligence (AI) tools are becoming part of how students think, not only what they use. Whether a learner engages with such a tool or delegates to it is a decision about how their own cognitive resources are deployed, which places that decision within the realm of study of human intelligence. Investment accounts of intellectual development hold that ability is built through motivated engagement with cognitively demanding material, yet technology acceptance frameworks emphasize utility judgments and underrepresent the motivational processes that drive such engagement. This study tests an extended Technology Acceptance Model in which self-efficacy shapes perceived usefulness through motivation and enjoyment. Survey data from 185 undergraduate School of Business students at a Saudi university who used generative AI tools during coursework were analyzed using partial least squares structural equation modeling. All nine hypotheses were supported. Self-efficacy strongly predicted both motivation and enjoyment, but motivation was the dominant mediating pathway to perceived usefulness (β = 0.295, *p* < 0.001), with enjoyment a weaker affective route (β = 0.086, *p* < 0.05), alongside a significant serial pathway from self-efficacy through motivation to enjoyment (β = 0.113, *p* < 0.001). The model explained 54.4% of the variance in behavioral intention. The findings indicate that beliefs about one’s own technical competence, rather than appraisal of the tool alone, govern how students commit to intelligent systems, with implications for whether AI use develops or displaces learners’ own intellectual capacity.

## 1. Introduction

In recent years, the integration of artificial intelligence (AI) into higher education has grown. This progress opens new opportunities for personalized and data-driven learning environments. AI-powered platforms, such as intelligent tutoring systems and recommendation engines, seek to enhance student engagement, provide automated feedback, and improve academic results (Chen et al., 2020; Ma et al., 2024). Understanding how students use and adopt these new tools has become crucial as universities worldwide embrace digital transformation (Mohamed Hashim et al., 2022).

The question this raises is not only which tools students will use, but what their use does to the learners themselves. Every decision to engage with an AI system rather than delegate to it is a decision about how a student’s own cognitive resources are allocated, and investment accounts of intellectual development hold that ability is built through the sustained, motivated application of those resources to demanding material (Cattell, 1963; Ackerman, 1996; von Stumm & Ackerman, 2013). Recent evidence bears this out in AI settings. The cognitive benefits of generative AI depend heavily on learners’ self-regulatory capacity (Zhao et al., 2025), while unguided reliance can produce a pattern of metacognitive laziness in which learners offload monitoring and evaluation to the system rather than performing it themselves (Fan et al., 2025). The resulting tension is further amplified by students’ own mixed perceptions regarding the feasibility and practical dilemmas of implementing generative AI in higher education (Sarwar et al., 2026). What disposes students toward engagement rather than delegation is therefore a question about human intelligence and how it develops, not only about technology acceptance.

While the technical potential of AI in education is clear, its success depends on students’ willingness to accept and consistently use it. The Technology Acceptance Model (TAM) is widely used to explain adoption in educational settings (Marikyan et al., 2023; Salloum et al., 2019), yet its core constructs of perceived usefulness and ease of use may not fully capture the emotional and cognitive factors that drive engagement with intelligent and adaptive tools (Holden & Rada, 2011; Ifinedo, 2017).

Recent studies have emphasized the importance of including emotional and cognitive factors, such as self-efficacy, motivation, and enjoyment, in expanded TAM frameworks (Naveed et al., 2023; Dehghani & Mashhadi, 2024). Students who feel comfortable using AI systems are more likely to be motivated, enjoy the experience, and perceive these tools as highly useful (Chiu et al., 2024; Yuan & Liu, 2025). Some research indicates that enjoyment and motivation are stronger predictors of technology adoption than perceived usefulness alone (Van der Heijden, 2004; Ursavaş, 2022). These findings highlight the need to move beyond traditional TAM concepts to better understand the complex factors driving AI adoption in higher education.

Three major research gaps motivate this study. First, while recent work emphasizes integrating self-efficacy, motivation, and enjoyment into TAM frameworks (Naveed et al., 2023; Dehghani & Mashhadi, 2024), few studies have systematically examined their serial-parallel mediating pathways, specifically whether self-efficacy influences perceived usefulness through both motivational and hedonic routes simultaneously, as well as cascading sequentially between them. Understanding these interconnected mechanisms is crucial for designing interventions that leverage cognitive confidence to enhance both intrinsic motivation and emotional engagement (Chiu et al., 2024). Second, existing TAM extensions mainly focus on general educational technologies rather than AI-powered platforms with adaptive and personalized features, which pose unique psychological demands due to their intelligent and responsive nature (Chen et al., 2020; Ma et al., 2024). Students’ acceptance of systems that dynamically modify content may differ from static learning tools, yet this difference remains underexplored. Third, empirical evidence primarily centers on Western and East Asian contexts (Holden & Rada, 2011; Marikyan et al., 2023), leaving Middle Eastern educational environments, especially the Gulf region, significantly underrepresented despite considerable national investments in digital transformation and AI adoption (Mohamed Hashim et al., 2022).

This study addresses these gaps by developing and testing an extended TAM that incorporates self-efficacy, motivation, enjoyment, and perceived usefulness to predict students’ intention to use AI-powered learning tools among undergraduates at a Saudi Arabian university, providing empirical evidence from an underrepresented regional context. In doing so, the study treats self-efficacy not as one more external variable appended to TAM, but as a tool-specific appraisal of one’s own competence (Flavell, 1979), and it treats intention to use as an indicator of a learner’s willingness to invest cognitive effort in an intelligent system. The research addresses the following research objectives (ROs):

**RO 1**—To investigate the mediating role of motivation in the relationship between self-efficacy and the perceived usefulness of AI-powered learning platforms;

**RO 2**—To examine how enjoyment mediates the relationship between self-efficacy and the perceived usefulness of AI-powered learning platforms;

**RO 3**—To evaluate the model’s fit, reliability, and predictive relevance for the adoption of AI tools in the higher education context.

## 2. Literature Review and Theoretical Background

A growing body of empirical research on AI tool adoption in higher education has moved beyond traditional technology acceptance models to integrate cognitive, affective, and contextual factors. This shift reflects the recognition that technology acceptance involves not only utilitarian judgments but also user confidence, motivation, and emotional engagement. Table 1 synthesizes key findings from the emerging literature.

In Saudi Arabia, extended Technology Acceptance Model (TAM) frameworks have revealed that factors such as knowledge, engagement, and motivation significantly influence perceived usefulness and behavioral intentions (Alharbi, 2025). Simultaneously, broader studies indicate positive stakeholder attitudes, although concerns regarding privacy and institutional preparedness remain prevalent (Al-Zahrani & Alasmari, 2024). In other regions, enjoyment has emerged as a strong predictor of AI tool adoption, occasionally exerting a greater influence than perceived usefulness, as evidenced by research on generative AI in Peruvian and Malaysian settings (Cano & Nunez, 2024; Dahri et al., 2024). Additionally, personalization enhances the intention to use AI platforms (Abdalla, 2024), although perceived usefulness remains a fundamental component of technology acceptance (Koteczki & Balassa, 2025). Overall, the evidence points to the growing recognition of psychological, affective, and contextual factors, such as motivation, enjoyment, trust, and personalization, in facilitating AI adoption, thereby affirming the relevance of the extended TAM to higher education research.

To guide such investigations, several theoretical models have been developed to understand technology adoption in educational contexts. Among the most prominent frameworks, the TAM has been widely adopted to examine user acceptance in educational settings, offering a robust framework for explaining and predicting technology usage behaviors (Davis, 1989; Venkatesh et al., 2003). However, traditional TAM constructs, namely, perceived usefulness and perceived ease of use, may not fully capture the motivational, emotional, and cognitive factors involved in students’ engagement with intelligent and adaptive technologies like AI-powered platforms (Alsubaie et al., 2025; Racero et al., 2020). This has prompted researchers to extend TAM by incorporating additional variables such as self-efficacy, motivation, and enjoyment, which are particularly relevant for evaluating the adoption of emerging technologies in learning environments (Abdullah & Ward, 2016; K. Cheng & Tsai, 2020; Huang & Teo, 2020).

### 2.1. Conceptual Model and Research Hypotheses

**Figure 1:**
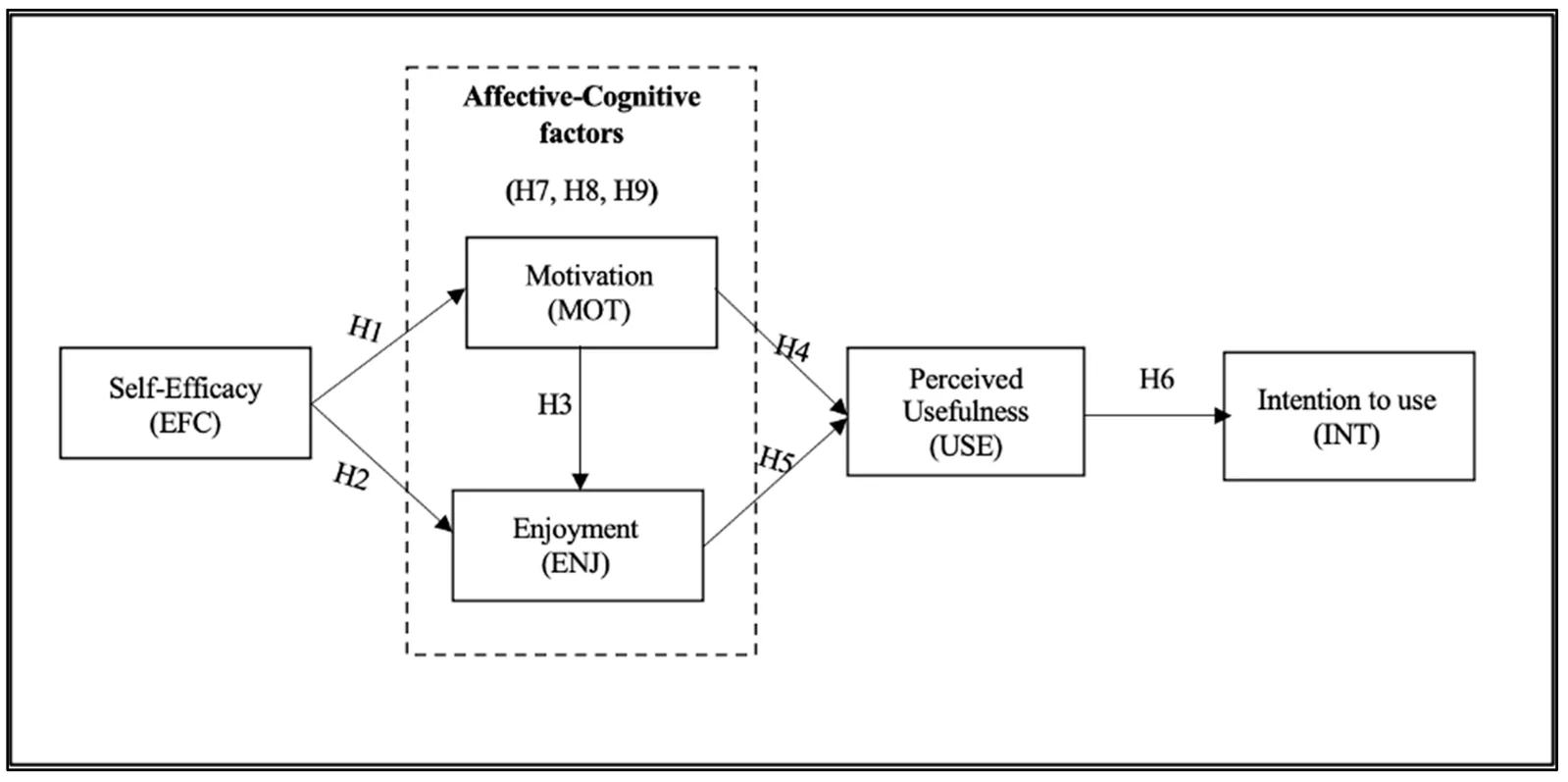
The research model.

The research model posits self-efficacy (EFC) as the foundational cognitive antecedent that influences perceived usefulness through two affective–cognitive mediators: motivation (MOT) and enjoyment (ENJ). Perceived usefulness (USE) in turn predicts behavioral intention to use the AI-powered learning platform (INT). This configuration enables the testing of six direct effects (H1–H6), as illustrated in Figure 1, along with three mediated pathways (H7–H9) representing the indirect effects of self-efficacy on perceived usefulness through motivation, through enjoyment, and sequentially through both.

#### 2.1.1. Self-Efficacy in Technology Adoption

Self-efficacy, grounded in Bandura’s Social Cognitive Theory, refers to individuals’ belief in their ability to successfully perform specific tasks (Bandura, 1997). In educational technology adoption, it reflects students’ confidence in using new tools to support their learning, significantly shaping acceptance, persistence, and continued use (Abdullah & Ward, 2016; Al-Rahmi et al., 2018; K. Cheng & Tsai, 2020).

Extensive empirical research highlights self-efficacy as a vital cognitive determinant of students’ intentions and behaviors in technology adoption (K. Cheng & Tsai, 2020; Zhai et al., 2021). For instance, W. Xu et al. (2025) found that self-efficacy significantly influenced perceived usefulness and behavioral intention to use a map-based online learning system, reinforcing its direct predictive power within an extended TAM framework.

Self-efficacy also influences users indirectly through mediating variables such as enjoyment and motivation. Functioning in a dual technical-motivational role, this perception fosters students’ intrinsic drive to engage with the technology (Bandura, 1997). Students who feel competent in technology use experience enhanced intrinsic motivation and enjoyment, which subsequently fosters more positive attitudes toward technology use and higher perceptions of usefulness (Abdullah & Ward, 2016; Granić, 2022; Pan, 2020).

Despite extensive research on self-efficacy in general educational technology contexts, few studies have explicitly explored its role in specialized AI-powered learning tools. Thus, investigating self-efficacy within the context of AI adoption provides an opportunity to address existing theoretical gaps and deepen understandings of learners’ acceptance behaviors (Al-Rahmi et al., 2018).

**H1.**  *Self-efficacy has a positive effect on students’ motivation to use AI-powered learning tools.*

**H2.**  *Self-efficacy has a positive effect on students’ enjoyment when using AI-powered learning tools.*

#### 2.1.2. Motivation as a Determinant of Technology Acceptance

Motivation plays a central role in shaping students’ engagement with educational technologies and their intention to adopt them. Intrinsic motivation, driven by internal satisfaction and curiosity, is associated with deeper cognitive engagement, long-term learning outcomes, and greater persistence (Ryan & Deci, 2000), while extrinsic motivation, driven by external rewards or pressures such as grades or praise, can be effective when intrinsic motivation is low but may not foster lasting engagement (Z. Zhou & Zhang, 2024). In AI-powered learning environments, motivation is particularly essential for sustaining user engagement and meaningful learning experiences.

Within extended versions of the Technology Acceptance Model (TAM), motivation has consistently emerged as a significant predictor of both perceived usefulness and behavioral intention. Empirical studies have shown that when motivation is integrated into TAM frameworks, it often explains a larger proportion of variance in intention to use technology than traditional TAM constructs alone (Lai et al., 2023). Motivated learners are more likely to perceive educational tools as valuable and aligned with their goals, enhancing their adoption decisions.

Research in educational technology also shows that motivation positively influences students’ enjoyment when using AI-powered learning tools. For instance, students using AI-powered platforms like Duolingo and ChatGPT reported significantly higher levels of both motivation and enjoyment compared to those using traditional methods, with increased motivation closely associated with greater enjoyment during learning activities (J. Xu & Liu, 2025; Yuan & Liu, 2025).

Motivation frequently mediates the relationship between self-efficacy and perceived usefulness. When individuals are confident in their ability to use a system, they are more intrinsically motivated to engage with it, which enhances their perception of its usefulness. In mathematics education, for example, self-efficacy has been shown to enhance intrinsic motivation, which in turn elevates perceived utility through a partial mediation pathway (Amjad et al., 2025). Similarly, in online language learning, strong self-efficacy encourages metacognitive strategy use, bolstering learning motivation and leading to higher perceptions of progress and usefulness (Teng & Wu, 2024).

Despite its recognized importance, the mediating role of motivation in the adoption of AI-powered educational technology remains underexplored, particularly in the higher education sector. Addressing this gap offers opportunities to extend current theoretical understanding and clarify the underlying mechanisms behind students’ acceptance and continued use of emerging AI-based tools.

**H3.**  *Motivation has a positive effect on students’ enjoyment in using AI-powered learning tools.*

**H4.**  *Motivation has a positive effect on students’ perceived usefulness of AI-powered learning tools.*

#### 2.1.3. Enjoyment in Educational Technology Adoption

Perceived enjoyment refers to the extent to which using a system is intrinsically pleasurable, independent of performance outcomes (Park et al., 2012). It consistently exerts a positive influence on perceived usefulness: when students find a learning tool enjoyable, they are more likely to view it as academically beneficial, and in some studies, perceived enjoyment has outperformed perceived usefulness as an adoption predictor (Kim et al., 2025).

Enjoyment also mediates the relationship between self-efficacy and perceived usefulness. Students confident in their ability to use AI tools are more likely to find them pleasurable, thereby elevating perceived usefulness (Duy et al., 2024). Chao (2019) confirmed that enjoyment mediated the relationships among self-efficacy, satisfaction, and sustained intention to use virtual learning environments, highlighting its significant indirect role in shaping user perceptions and continued usage.

Despite the growing recognition of enjoyment’s importance, research on its mediating role specifically within AI-powered educational tools remains relatively sparse. Given AI’s ability to enhance personalized, adaptive, and enjoyable learning experiences, exploring the mediating role of enjoyment in AI-enhanced educational contexts is an essential contribution to the existing TAM literature.

**H5.**  *Enjoyment has a positive effect on students’ perceived usefulness of AI-powered learning tools.*

#### 2.1.4. Perceived Usefulness of AI-Powered Learning Tools

Perceived usefulness, originally articulated within the Technology Acceptance Model (TAM), represents users’ subjective beliefs regarding how effectively a technology enhances their job or learning performance. Within educational settings, perceived usefulness significantly influences students’ attitudes, intentions, and sustained usage of educational technologies, acting as a primary determinant of adoption decisions (Scherer & Teo, 2019; Huang & Teo, 2020).

Empirical evidence consistently supports perceived usefulness as the strongest predictor of behavioral intentions toward educational technology adoption. Scherer and Teo (2019) concluded that perceived usefulness outperformed other factors, including perceived ease of use and attitude, in predicting students’ behavioral intentions across diverse educational technologies, while Huang and Teo (2020) similarly found it critical to both initial adoption and ongoing engagement with digital learning resources.

In the context of AI-powered learning tools, perceived usefulness takes on particular importance due to AI’s ability to personalize learning, automate feedback, and dynamically adapt content to student needs (Soliman et al., 2025; Zhai et al., 2021). For example, Zhai et al. (2021) emphasized that students’ perceptions of usefulness were positively enhanced by AI-driven adaptive systems, leading to increased motivation, engagement, and academic achievement. Similarly, several studies demonstrated that AI-enhanced feedback significantly improved students’ perceptions of learning outcomes, reinforcing their intentions to adopt and continuously use AI-based learning platforms (Qiu, 2025; Shuaibu et al., 2024).

Moreover, perceived usefulness frequently serves as a mediator, translating motivational and affective experiences, such as intrinsic motivation and enjoyment, into tangible technology adoption outcomes (Huang & Teo, 2020; Racero et al., 2020). The evidence suggests that students’ intrinsic motivation positively influences perceived usefulness, subsequently shaping their intention to adopt digital learning platforms, reinforcing its central role within extended TAM frameworks.

Despite the broad recognition of perceived usefulness within general educational technologies, targeted studies explicitly addressing it in the context of AI-driven tools remain limited (Zhai et al., 2021).

**H6.**  *Perceived usefulness has a positive effect on students’ intention to use AI-powered learning tools.*

#### 2.1.5. Behavioral Intention Toward AI Use

Behavioral intention, a core construct in technology acceptance frameworks, refers to the likelihood that an individual will adopt and continue using a particular technology (Ma et al., 2024; Rejali et al., 2023; Scherer & Teo, 2019). In educational contexts, it is widely regarded as the strongest predictor of actual technology use (Alsubaie et al., 2025; M. Cheng & Yuen, 2018).

Prior research confirms its robust predictive capacity: Teo and Zhou (2017) demonstrated that behavioral intention significantly predicted actual educational technology usage across diverse contexts, while Almaiah and Alismaiel (2019) showed that students’ intentions were substantially shaped by their perceptions of usefulness and ease of use (Teo & Zhou, 2017; Almaiah & Alismaiel, 2019).

In AI-powered educational contexts specifically, perceived usefulness, personalization, and interactivity significantly enhance adoption intentions (Hwang & Xie, 2018), and behavioral intention has been shown to mediate between AI technology features and their effective integration in higher education (Zawacki-Richter et al., 2019).

Behavioral intention is also an outcome of the affective–cognitive mediators central to this study. Huang et al. (2021) confirmed that perceived usefulness, driven by intrinsic motivation and enjoyment, significantly enhanced students’ intentions to use adaptive educational technologies, with prior work further supporting motivation and enjoyment as mediation paths to behavioral intention (Jeno et al., 2017; Rosli & Saleh, 2023). Despite this evidence, the explicit investigation of behavioral intentions toward AI-powered learning tools specifically remains limited, motivating the mediation analysis in the following section.

#### 2.1.6. The Mediating Role of Affective–Cognitive Factors: Integrating Motivation and Enjoyment

Affective–cognitive factors, such as enjoyment and motivation, play a crucial role in shaping individuals’ attitudes and subsequent behaviors across various contexts, including technology adoption and learning environments. According to Lavine et al. (1998), affect, comprising emotional responses, can exert primacy over cognition in influencing attitudes and behaviors, especially when affective–cognitive ambivalence exists. Enjoyment, as an intrinsic affective factor, enhances engagement by fostering positive emotional experiences during interactions, which in turn can promote sustained usage and deeper learning (Acosta-Enriquez et al., 2024). Motivation, on the other hand, acts as a driving force that energizes goal-directed behaviors, often shaped by personal beliefs such as self-efficacy.

Self-efficacy, defined as an individual’s belief in their capacity to execute specific tasks successfully (Bandura, 1997), has been identified as a pivotal cognitive factor influencing motivation and affective responses. High self-efficacy enhances motivation by fostering confidence in one’s abilities, which motivates individuals to engage more actively with new technologies or tasks (Acosta-Enriquez et al., 2024). Additionally, enjoyment arises from the positive emotional experiences associated with task engagement, which can be intensified by a strong sense of self-efficacy. Empirical research supports this link; J. Zhou et al. (2013) demonstrated that affect-based attitudes, such as enjoyment, are driven by cognitive evaluations like self-efficacy, and these, in turn, influence behavioral intentions.

Motivation, as an affective–cognitive factor, amplifies the effect of self-efficacy by fostering a proactive stance toward technology use, which enhances perceptions of its utility (Van Harreveld et al., 2015). For instance, individuals with high self-efficacy are more likely to be motivated to explore and utilize technological features, thereby perceiving the technology as more useful. Similarly, enjoyment provides positive reinforcement, making the task more pleasurable and reinforcing perceptions of usefulness. Empirical studies on attitude formation suggest that positive affective experiences, such as enjoyment, significantly mediate the influence of cognitive factors like self-efficacy on attitudes toward technology (Arvola et al., 2008). Therefore, both motivation and enjoyment are critical pathways through which self-efficacy translates into perceptions of usefulness, with motivation sustaining engagement and positive emotional experiences particularly in challenging tasks (Lavine et al., 1998).

Importantly, these mechanisms are not conceptualized solely as parallel mediators. Given the proposed relationship between motivation and enjoyment (H3), the model also incorporates a serial pathway linking self-efficacy to perceived usefulness sequentially through motivation and enjoyment (H9). Specifically, stronger self-efficacy can foster greater motivation to engage with and explore AI technologies; this heightened motivation may, in turn, facilitate more positive and enjoyable experiences during technology use, ultimately strengthening perceptions of usefulness. This sequential mechanism is consistent with the notion that motivation can sustain engagement and facilitate positive affective experiences, particularly when individuals interact with challenging tasks or technologies (Lavine et al., 1998). Accordingly, the model distinguishes three theoretically meaningful indirect pathways: self-efficacy through motivation to perceived usefulness, self-efficacy through enjoyment to perceived usefulness, and a serial pathway from self-efficacy through motivation and enjoyment to perceived usefulness. Based on this reasoning, the following hypotheses are proposed:

**H7.**  *Motivation significantly mediates the relationship between self-efficacy and perceived usefulness, such that higher self-efficacy leads to increased motivation, which in turn enhances perceived usefulness;*

**H8.**  *Enjoyment significantly mediates the relationship between self-efficacy and perceived usefulness, with higher self-efficacy fostering greater enjoyment, thereby increasing perceived usefulness;*

**H9.**  *Motivation and enjoyment sequentially mediate the relationship between self-efficacy and perceived usefulness, such that higher self-efficacy increases motivation, which subsequently enhances enjoyment and, in turn, increases perceived usefulness.*

### 2.2. Situating the Model Within Research on Human Intelligence

The constructs in this model are conventionally treated as antecedents of technology acceptance, but each has a longer history in research on intelligence and its development. Investment theories hold that measured ability is not a fixed capacity alone, but the accumulated product of how, and how persistently, individuals apply their cognitive resources. Cattell (1963) proposed that crystallized ability precipitates out of fluid ability invested in learning over time, and Ackerman (1996) formalized this in the PPIK framework, in which personality and interests direct the intellectual investment that yields knowledge. A meta-analytic review of investment constructs confirms that dispositions toward engaging with effort with intellectual material are reliably associated with ability outcomes (von Stumm & Ackerman, 2013). Motivation and enjoyment, as modeled here, are the proximal situational analogues of those dispositions; they describe whether a student will bring effort to bear on a given intellectual task at a given moment. Viewed through this lens, perceived usefulness represents more than a functional evaluation of technology; it signifies the perceived return on intellectual investment. As such, self-efficacy plays a dual cognitive–motivational role by acting both as a metacognitive appraisal of capability and as the foundational driver for effortful engagement.

Self-efficacy occupies a similarly established position. Beliefs about one’s own capability are metacognitive judgments—that is, cognition directed at one’s own cognitive processes (Flavell, 1979)—and their calibration has direct behavioral consequences. Risko and Gilbert (2016) show that the decision to offload a cognitive task to an external aid is governed substantially by metacognitive evaluations of one’s own mental abilities, and that these evaluations are frequently inaccurate, producing suboptimal offloading. Recent evidence extends this to generative AI. Across Turkish-speaking and English-speaking student samples, heavier AI use predicted weaker critical thinking dispositions, and that association was carried sequentially through metacognitive weakness and epistemic laziness rather than through exposure to the technology itself (Kırcaburun, 2026). Within the intelligence literature, academic self-efficacy has been shown to mediate the translation of situational demands into creative and cognitive outcomes (Yao et al., 2023). Self-efficacy in this model is therefore not simply a technology-related belief; it is the learner’s working model of their own competence, and it is what determines whether an intelligent tool is approached as a partner in thinking or as a substitute for it.

Framing the model this way clarifies what is at stake in AI adoption. Whether an intelligent tool contributes to cognitive development appears to depend on what the learner does with it rather than on exposure to it. Among 878 undergraduates, the association between AI cognitive stimulation and creative capacity ran almost entirely through learners’ own divergent thinking rather than through the AI-generated content itself (Al-khresheh et al., 2026). Meta-analytic evidence indicates that generative AI produces moderate gains in higher-order thinking overall, but that these gains are conditional on learners’ self-regulated learning capacity, with high-capacity learners benefiting roughly three times as much as low-capacity learners (Zhao et al., 2025). Experimental work points the same way from the opposite direction, showing that learners supported by generative AI may improve task output while disengaging from the metacognitive processes that produce durable learning (Fan et al., 2025). If adoption is driven by confidence and motivated engagement, it is more likely to build intellectual capacity; if it is driven mainly by the wish to reduce effort, it is more likely to erode it. The model tested here specifies which of these psychological routes is operative.

## 3. Research Methodology

This study employed a quantitative, survey-based approach to examine undergraduate students’ perceptions and intentions regarding the use of AI-learning educational tools in higher education. The target population consisted of students enrolled in a specific course within the School of Business at a large public university in Saudi Arabia. Participants were selected using convenience sampling based on their enrollment during the semester in which the study was conducted. During their coursework, these students were required to use generative AI tools such as ChatGPT to complete research and data analysis assignments.

Data were collected through a structured questionnaire. Prior to participation, students received a consent form explaining the voluntary nature of the study and assuring them of data confidentiality. The survey began with demographic questions covering gender, age, and self-rated computer and AI skill levels, followed by items measuring the key constructs of interest. No names, student identification numbers, or other direct identifiers were collected at any stage, and all responses were recorded anonymously. All items were assessed using a 7-point Likert scale, ranging from strongly disagree to strongly agree. The study protocol was reviewed and approved by the IRB to ensure participant protection and ethical compliance.

The measurement model was adapted from several well-established studies (see Table 2 for the full initial item pool) and reviewed by domain experts to ensure content validity and relevance. The Motivation scale was designed to capture both students’ internal drive and their perception of the tool’s empowering features, such as self-paced learning and user control, which are established antecedents to academic motivation. However, because this operationalization largely reflects system affordances and perceived empowerment rather than purely intrinsic drive, it introduces semantic overlap with both self-efficacy and perceived usefulness, a construct validity limitation that may partially inflate the structural pathways connecting these variables. Structural Equation Modeling (SEM) was conducted using SmartPLS, which implements Partial Least Squares (PLS) path modeling. This technique was chosen due to its suitability for exploratory studies and its ability to handle complex models with relatively small sample sizes.

The adequacy of the sample size was confirmed using the A-priori Sample Size Calculator for SEM. With an assumed effect size of 0.3, alpha level of 0.05, five latent variables, twenty observed variables, and statistical power of 0.80, the analysis recommended a minimum of 150 participants.

## 4. Results

This section presents the results from analyzing participant responses. Responses were downloaded and coded using Microsoft Excel. A dataset was created for further analysis by coding variables and removing records with missing data. As the questionnaire collected no direct identifiers, no de-identification step was required. A descriptive analysis was performed to show the frequencies and distributions of participants across computer and AI skill levels, as well as between male and female genders. Finally, the dataset was analyzed in SmartPLS version 3.3 for measurement model and structural model evaluation. The following sections detail the results.

### 4.1. Demographic Data

A total of 185 valid and complete responses show that 97 (52%) are male and 88 (48%) are female. The average age for both genders is close to 21 years, with maximum ages of 28 and 29, respectively. Table 3 depicts the statistics of age by gender.

Most participants (51%) fall into the intermediate category for computer skills, and 51% are in the beginner’s category for AI skills. Beginners in computer skills make up 39%, while 41% say they have an intermediate level of AI skills. The small percentages for both categories are at the extremes, having either no skills or advanced levels. Table 4 provides detailed frequencies and percentages. The descriptive statistics show that the sample is diverse in gender, age, computer skills, and AI skills.

### 4.2. Measurement Model Assessment

Following the guidelines for conducting measurement model analysis using SmartPLS, the model is evaluated for its validity, reliability, and discriminant validity using thresholds recommended by Hair et al. (2017); Henseler (2017); and Fornell and Larcker (1981). During initial assessment, two items (EFC1 and EFC4) demonstrated factor loadings below the 0.7 threshold and were removed to improve construct validity. While typically requiring three indicators, two-item constructs are considered acceptable in PLS-SEM when they are reflective and exhibit strong psychometric properties, as evidenced by the high factor loadings and composite reliability reported here. In this context, it should be noted that the removal of the aforementioned items narrowed the conceptual domain of the self-efficacy construct in the study, which allows for aligning it more closely with navigational and technical feature competence rather than broad academic or metacognitive confidence. The remaining items showed strong psychometric properties. The factor loadings for all items within their respective construct are above 0.7 (Hair et al., 2017). Similarly, the Cronbach’s alpha and composite reliability measure for all constructs are above 0.7 (Henseler, 2017). The average variance extracted value for all constructs is above 0.5, as recommended by (Fornell & Larcker, 1981), thus establishing the validity and reliability of the research model. Table 5 lists all related metrics, thresholds, and values.

Table 6 shows the square root of the AVE value of each construct being higher than the values for any other constructs, thus establishing the discriminant validity using the Fornell–Larcker method (Fornell & Larcker, 1981).

A second method of evaluating discriminant validity is by using the heterotrait–monotrait (HTMT) correlation ratio (Henseler et al., 2016). The threshold should not exceed the value of 0.9 for all constructs, which is established as indicated by Table 7.

### 4.3. Testing of the Structural Model and Hypotheses

**Figure 2:**
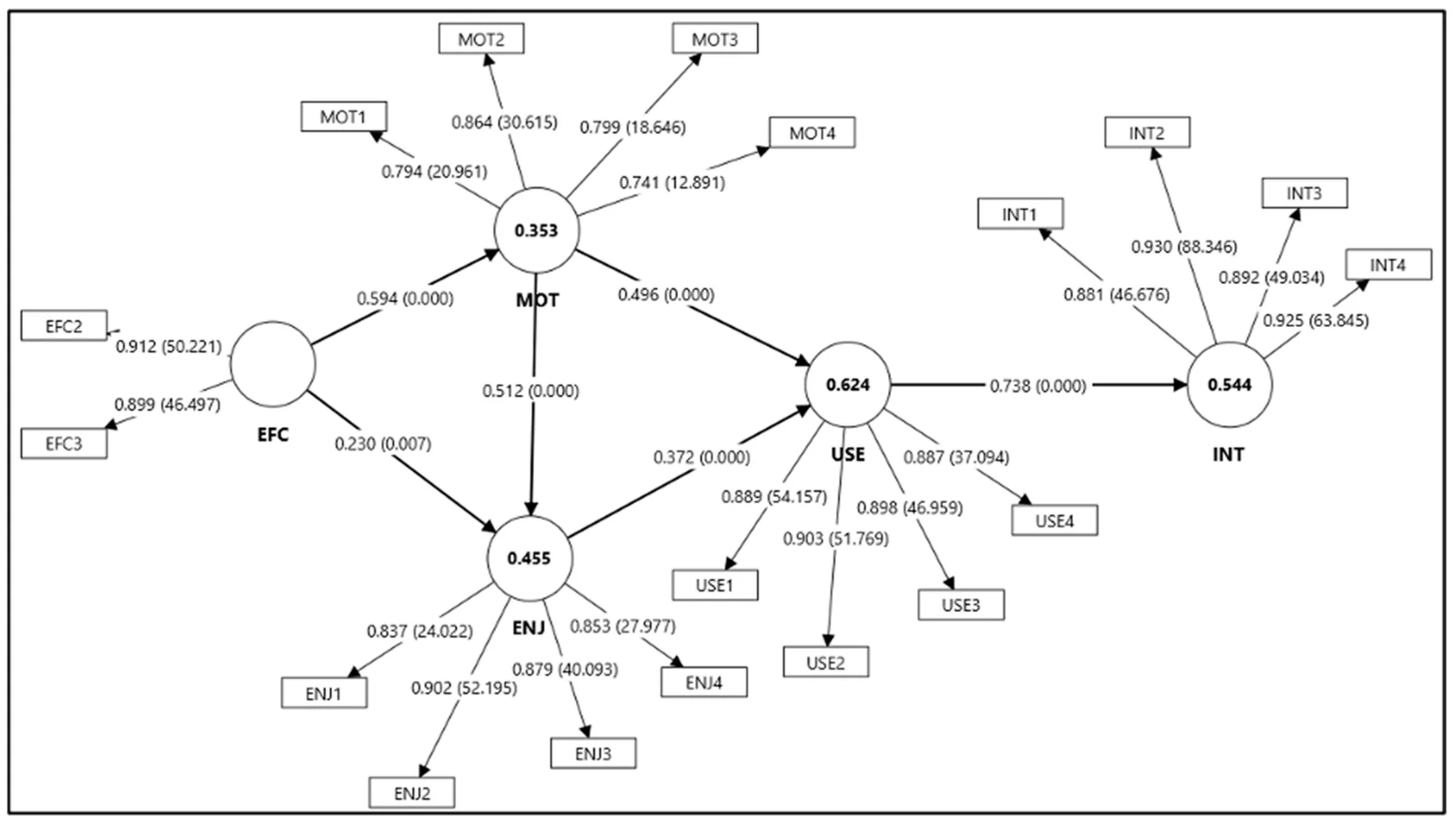
Graphical representation of the path coefficient.

The structural model analysis shows positive and significant effects on all direct paths indicated in Figure 2. Efficacy has a large effect (F2 = 0.546) on motivation (β = 0.594; *p* < 0.001) and a small effect (F2 = 0.063) on enjoyment (β = 0.230; *p* < 0.05). This supports both hypotheses 1 and 2. Additionally, motivation has a medium effect (F2 = 0.311) on enjoyment (β = 0.512; *p* < 0.001) and a large effect (F2 = 0.379) on usefulness (β = 0.496; *p* < 0.001), supporting hypotheses 3 and 4. Enjoyment has a medium effect (F2 = 0.214) on usefulness (β = 0.372; *p* < 0.001), and usefulness has a medium effect (F2 = 1.194) on the intention to use (β = 0.738; *p* < 0.001), supporting hypotheses 5 and 6. Table 8 provides details on the hypotheses and their related results from the path analysis.

The model specifies three indirect pathways from self-efficacy to perceived usefulness: two mediated separately by motivation and enjoyment, and one serial pathway running through both in sequence. All specific indirect effects were estimated using bootstrapping, with 5000 subsamples and bias-corrected and accelerated confidence intervals.

The pathway via motivation is significant and carries the largest indirect effect (β = 0.295, t = 5.389, *p* < 0.001), supporting H7. The parallel pathway via enjoyment is significant but substantially weaker (β = 0.086, t = 2.235, *p* = 0.025), supporting H8. The serial pathway implied by the model structure was formally estimated, in which self-efficacy raises motivation, motivation raises enjoyment, and enjoyment in turn raises perceived usefulness. This pathway is also significant (β = 0.113, t = 3.700, *p* < .001), supporting H9. The total effect of self-efficacy on perceived usefulness is therefore fully carried by the three indirect pathways and equals β = 0.494. Table 9 reports these results.

To determine the predictive effect, model relevance, and the average difference between observed and predicted correlations among variables, the R-squared, Q-squared, and SRMR values are calculated for the endogenous variables. Efficacy accounts for about 35% of the variation in motivation, while efficacy and motivation together explain 45.5% of the variation in enjoyment. Additionally, motivation and enjoyment explain 62.4% of the variation in usefulness. Usefulness accounts for 54.4% of the variance in the intention to use. These R-squared values indicate moderate predictive power according to Chin (1998), but a substantial effect according to Cohen (1988). Furthermore, the predictive relevance of the model is confirmed by Q-squared values above 0 for all endogenous variables. Finally, the Standard Root Mean Squared Residual (SRMR) is 0.075, which is below the threshold of 0.08, indicating good fit for the model used in this study. Table 10 presents the calculated values for R^2^, Q^2^, and SRMR.

## 5. Discussion

This study investigated the factors influencing students’ acceptance of AI-powered learning tools in higher education by developing and testing an extended Technology Acceptance Model. The analysis supported all hypothesized relationships (H1–H9), revealing significant insights into how cognitive confidence (self-efficacy) influences perceived usefulness through affective–cognitive mediators (motivation and enjoyment), ultimately shaping behavioral intentions. The following discussion interprets these findings in relation to each research objective.

### 5.1. Findings in the Regional Context

One of the main insights emerging in the analytical stage pertains to motivation, as a driver of AI adoption, playing the predominant role over enjoyment. This finding can be interpreted through the lens of the regional higher education context. Spurred by national digital transformation strategies across the Gulf region, universities have actively integrated digital transformation into their strategic goals, while prioritizing academic performance and technological competency (Mohamed Hashim et al., 2022). The newly formed top-down policy environment fosters a highly pragmatic educational culture. Where adoption is directed institutionally rather than emerging from individual experimentation, technologies tend to arrive with an official purpose already attached, encountered first as means to defined academic outcomes rather than as objects of open exploration. A policy agenda that frames technological competency as a national priority gives students corresponding reason to treat proficiency with AI tools as an expectation to be met rather than an interest to be pursued. Under such circumstances, students are likely to perceive AI tools through a highly instrumental lens due to their function as essential assets for meeting institutional expectations and securing future employability. This perspective contrasts with an often-exaggerated position of AI solutions as nearly exclusively a source of leisure or entertainment. As the associated contextual pressure amplifies the role of motivation, driven by achievement goals as well as academic requirements, and dampens the relative importance of enjoyment, it is expected that confidence in using these tools may translate more strongly into purposeful engagement than into hedonic pleasure.

### 5.2. The Mediating Role of Motivation Between Self-Efficacy and Perceived Usefulness

Motivation emerged as the dominant mediating pathway (β = 0.295, *p* < 0.001); students with greater confidence in their AI skills reported higher motivation, which in turn enhanced perceptions of usefulness. This finding aligns with prior research demonstrating that self-efficacy serves as a foundation for intrinsic motivation in technology adoption contexts (K. Cheng & Tsai, 2020; Abdullah & Ward, 2016). The large effect size of self-efficacy on motivation (f^2^ = 0.546) underscores its pivotal role in fostering motivational engagement with AI tools. The observed strong motivational pathway aligns with Alharbi (2025), who also found motivation to be a key predictor of perceived usefulness in a Saudi context. Theoretically, this mediation relationship extends TAM by revealing motivation as a critical mechanism through which cognitive confidence translates into evaluative judgments about technology usefulness. Students who feel competent using AI systems develop a stronger internal drive to engage with these tools, subsequently recognizing their academic value more readily. Nevertheless, it is critical to acknowledge that the study’s motivation construct strongly captured perceptions of system affordances, such as self-paced learning and user control. As a result, the established dominant pathway reflects students’ sense of technical empowerment rather than purely intrinsic drive, whereas the semantic overlap between these empowerment affordances, self-efficacy, and perceived usefulness may partially inflate the observed structural coefficients. This pathway is particularly relevant for AI-powered platforms, which require sustained engagement to realize their adaptive and personalized capabilities (Ryan & Deci, 2020).

### 5.3. The Mediating Role of Enjoyment Between Self-Efficacy and Perceived Usefulness

The enjoyment pathway was also significant (β = 0.086, *p* < 0.05), though notably weaker. Students with higher self-efficacy experienced greater enjoyment, which contributed to enhanced perceptions of usefulness. This finding corroborates previous research identifying enjoyment as a relevant affective factor in educational technology acceptance (Duy et al., 2024; Chao, 2019). Nevertheless, it contrasts with findings from other cultural contexts, such as Cano and Nunez (2024) in Peru, where enjoyment was the primary driver of intention. In this context, the preeminence of utilitarian over hedonic factors may have played a decisive role in our specific academic setting.

However, the smaller effect size warrants careful interpretation. While self-efficacy does foster enjoyment, this relationship (β = 0.230, f^2^ = 0.063) is substantially weaker than its effect on motivation. This suggests that confidence in one’s technical ability does not automatically translate into hedonic pleasure during system use. Considering that motivation was found to be a strong predictor of enjoyment (H3, β = 0.512), our results point to a cascading psychological effect, quantitatively supported by the serial pathway as illustrated in Table 9, where self-efficacy primarily builds motivation, which in turn fosters enjoyment, rather than these two factors operating as strictly parallel or independent pathways. Students may feel competent using AI tools without necessarily finding them inherently enjoyable, particularly when these tools are perceived primarily as instrumental aids rather than intrinsically pleasurable experiences. This distinction may be especially relevant for AI platforms deployed in formal educational settings, where utilitarian goals (completing assignments, improving grades) often overshadow hedonic motivations.

The differential strength of these two mediation pathways highlights the importance of incorporating multiple affective–cognitive constructs in technology acceptance models. Both motivation and enjoyment contribute to perceived usefulness, but they operate through distinct psychological mechanisms and are not interchangeable. In this context, active and motivated empowerment appears to be significantly more critical for sustained cognitive engagement than passive enjoyment.

### 5.4. Model Fit, Reliability, and Predictive Relevance

The extended TAM demonstrated strong psychometric properties and predictive power. All factor loadings exceeded 0.74, with composite reliability and AVE values above established thresholds (Hair et al., 2017); discriminant validity was confirmed via Fornell–Larcker and HTMT criteria, empirically establishing that self-efficacy, motivation, and enjoyment function as distinct psychological constructs rather than redundant pathways (Acosta-Enriquez et al., 2024). In terms of explanatory power, self-efficacy accounted for 35.3% of variance in motivation, indicating that confidence is a substantial but not exclusive driver, as institutional support and prior experience may also contribute (Marasinghe et al., 2024). Self-efficacy and motivation jointly explained 45.5% of variance in enjoyment, reflecting that affective responses stem largely from competence beliefs and perceived empowerment (Ryan & Deci, 2020). Most notably, motivation and enjoyment together explained 62.4% of variance in perceived usefulness, underscoring that usefulness judgments are shaped as much by affective–cognitive engagement as by rational appraisal (K. Cheng & Tsai, 2020), and the model ultimately explained 54.4% of variance in behavioral intention (Ma et al., 2024). Predictive relevance was confirmed by Q^2^ values of 0.151–0.337 (Chin, 1998), and model fit was acceptable (SRMR = 0.075; Henseler et al., 2016), validating the model’s applicability to broader Gulf-region higher education contexts and beyond.

### 5.5. Implications for Research on Human Intelligence

Read through the lens of intellectual investment, the pattern of coefficients is informative. Self-efficacy exerted a large effect on motivation (f^2^ = 0.546), but only a small one on enjoyment (f^2^ = 0.063), and motivation carried roughly three and a half times as much of the indirect effect on perceived usefulness as enjoyment did (β = 0.295 versus β = 0.086). Students who believe they are competent with these systems do not primarily find them pleasurable; they find them worth working with. That is the signature of instrumental investment rather than hedonic consumption, and the distinction matters for intelligence research because the two dispositions carry different cognitive consequences. Effortful, goal-directed engagement is the mechanism by which fluid capacity is converted into durable knowledge (Cattell, 1963; Ackerman, 1996); engagement sustained mainly by enjoyment offers no equivalent guarantee. The weak hedonic pathway is also consistent with evidence that affect in human–AI collaboration has a narrower cognitive reach than is often assumed; positive affective experience during AI-assisted work has been found to arise from the social texture of the interaction rather than from its cognitive support, and to be associated with convergent but not divergent thinking (Y. Cheng & Huang, 2026).

This permits a more optimistic reading of AI adoption in this sample than the cognitive offloading literature might predict. Were students adopting these tools chiefly to reduce cognitive demand, self-efficacy should be a weak or even negative predictor of engagement, since offloading is most attractive to those who doubt their own capability (Risko & Gilbert, 2016). The observed pattern is the reverse: confidence in one’s technical and navigational competence is what drives engagement, which is consistent with tool use that supplements rather than replaces the learner’s own processing. The caution is that this study measured behavioral intentions as psychological antecedents of adoption, rather than actual cognitive or metacognitive outcomes. Whether motivated adoption actually yields gains in higher-order thinking depends on learners’ self-regulatory capacity (Zhao et al., 2025) and on whether the interaction preserves the metacognitive work that learning requires (Fan et al., 2025). The risk is not hypothetical: heavier AI use among university students has been associated with weaker critical thinking dispositions, carried through metacognitive weakness rather than through usage volume alone (Kırcaburun, 2026). This observation aligns with recent evidence highlighting the practical dilemmas students face between the educational feasibility of generative AI and the risks of unguided reliance (Sarwar et al., 2026). Establishing that link is the natural next step for this line of research.

## 6. Conclusions

### 6.1. Theoretical Implications

This study advances technology acceptance theory by integrating affective (enjoyment) and cognitive (motivation, self-efficacy) dimensions within TAM, addressing critiques that traditional TAM neglects emotional factors. Critically, we demonstrate that motivation and enjoyment function as mediators rather than direct predictors, clarifying how self-efficacy translates into perceived usefulness. This dual contribution, construct integration and causal pathway refinement, moves TAM beyond incremental extensions toward a mechanistic explanation. Our research differs from prior affective TAM extensions in three ways. First, rather than treating enjoyment as an additional predictor, we theorize that it serves as a mediating mechanism, explaining the psychological process linking self-efficacy to usefulness perceptions. Second, we examine dual mediation pathways simultaneously, revealing differential strengths, which suggests that motivation is the primary mechanism. Third, we contextualize these relationships within AI-powered learning environments, which present unique psychological challenges compared to general educational technologies.

Beyond technology acceptance, these findings speak to a question in intelligence research: what disposes learners to invest cognitive effort in intelligent systems rather than delegate to them? By showing that a technical self-appraisal drives engagement predominantly through a motivational rather than a hedonic route, the model identifies confidence in one’s own tool-specific competence as the point at which AI use is most likely to become intellectually productive. Importantly, such adoption intentions reflect psychological precursors rather than realized cognitive outcomes. From this perspective, technology acceptance research is positioned as one input to the study of how ability develops under technologically mediated conditions, alongside recent work modeling the cognitive processes that AI use engages or bypasses (Al-khresheh et al., 2026; Kırcaburun, 2026), rather than as a separate branch of the literature.

### 6.2. Practical Implications

The findings carry practical implications for multiple stakeholders. University administrators should invest in training programs and technical support to build student self-efficacy, while extending adoption metrics beyond usage rates to include motivation and satisfaction. Furthermore, institutions could implement targeted AI literacy workshops to systematically teach students how to leverage such tools for cognitive extension rather than passive offloading. Platform designers should balance utilitarian and hedonic qualities by integrating interface cues that prompt deliberate cognitive effort alongside interface fluidity, gamification, and personalization options to build both confidence and enjoyment. Instructors should integrate AI tool training into course orientation, model effective usage during instruction, and design assignments that require meaningful rather than optional engagement. For example, scaffolded tasks could require students to critically evaluate AI-generated outputs against primary scientific literature. Students should recognize that early discomfort with AI tools typically reflects unfamiliarity rather than inadequacy, and that sustained exploration builds the intrinsic motivation our findings identify as the primary pathway to technology acceptance.

### 6.3. Limitations and Future Research Directions

Several limitations should be noted. The cross-sectional design limits causal inference; longitudinal studies would better capture how self-efficacy, motivation, and enjoyment develop with continued AI use. Convenience sampling within a single faculty at one Gulf-region institution constrains generalizability, and reliance on self-report measures may introduce common method bias; future work should employ multi-site comparisons, use probability sampling across disciplines, and supplement surveys with behavioral usage data to establish behavioral links. In terms of construct validity, the self-efficacy construct was retained with two items due to low loadings on two others, which inadvertently narrowed the conceptual domain to navigational and technical competence rather than broader intellectual confidence. Similarly, the motivation scale to a great extent captured system affordances and perceived learner control, creating semantic overlap with both self-efficacy and perceived usefulness that may have artificially inflated the strength of these structural pathways; therefore, more comprehensive and distinctly validated scales are recommended.

Cross-cultural studies are needed to determine whether mediation pathways function similarly in other contexts, and future models should explore potential moderators (age, gender, discipline, prior experience), and socio-ethical factors such as trust, data privacy concerns, and algorithmic transparency, which may substantially shape AI acceptance beyond traditional TAM constructs. A further limitation is that the study measured behavioral intention rather than cognitive outcomes, so the argument that motivated adoption supports intellectual development remains an inference from the pattern of pathways rather than a demonstrated effect; testing it would require pairing this model with direct measures of higher-order thinking, or with instruments designed to capture metacognitive regulation during AI use such as the Strategic Prompting Scale (Suriano & Plebe, 2026).

Despite these limitations, this study offers strong empirical evidence for the expanded TAM in AI-powered learning settings, and shows that cognitive confidence, motivational factors, and pleasure collectively influence students’ acceptance of educational AI. As universities worldwide increasingly incorporate artificial intelligence into teaching and learning, understanding these psychological factors is crucial for designing effective, student-focused implementations that promote real engagement rather than just surface-level compliance. By illustrating how self-efficacy affects technology acceptance through motivational and emotional pathways, this research provides both theoretical insight and practical guidance for the ongoing digital transformation in higher education.

## Figures and Tables

**Table 1:** Recent empirical studies on AI tool adoption in higher education.

Study	AI Tool/Context	Country	Framework	Key Finding
Alharbi (2025)	AI tools for EFL learning	Saudi Arabia	Extended TAM (knowledge, motivation)	Perceived knowledge, engagement, and motivation significantly predicted perceived usefulness (PU) and adoption intentions.
Al-Zahrani and Alasmari (2024)	General AI in HE	Saudi Arabia	Descriptive stakeholder survey	Strong positive attitudes toward AI; emphasis on ethics, personalization, and institutional readiness.
Cano and Nunez (2024)	Generative AI in innovation courses	Peru	TAM variant (enjoyment focus)	Perceived enjoyment strongly predicted intention to use GenAI; PU had no significant effect.
Dahri et al. (2024)	ChatGPT in pre-service teacher education	Malaysia	Extended TAM (incl. enjoyment, trust)	Enjoyment, trust, perceived AI usefulness, and personal competency significantly predicted acceptance.
Abdalla (2024)	ChatGPT adoption intentions	Global sample (incl. Gulf context)	TAM + personalization moderator	Personalization increased intention to use ChatGPT among students.
Koteczki and Balassa (2025)	AI tools adoption among Gen Z	Likely Poland/Europe	Extended TAM/UTAUT hybrid	Perceived usefulness remained the most critical predictor of AI adoption.

**Table 2:** Variables and items included in the questionnaire.

Variable	Items	Statement	Reference
Self-Efficacy	EFC1	If I encounter problems while using AI tools, I can find solutions.	Ahn (2024).
	EFC2	I can quickly learn how to use new features of AI tools.	
	EFC3	I am capable of independently navigating AI tools.	
	EFC4	I feel competent using AI tools in my academic work.	
Motivation	MOT1	I believe I can use AI tools effectively.	Sheldon and Gunz (2009)
	MOT2	I am certain I can use AI tools to enhance my learning.	
	MOT3	AI tools enable self-paced learning.	
	MOT4	AI tools provide a sense of control over my learning.	
Enjoyment	ENJ1	Using AI tools puts me in a positive mood.	Cui (2025)
	ENJ2	I find using AI tools enjoyable.	
	ENJ3	Using AI tools makes learning fun.	
	ENJ4	I find learning with AI tools to be interesting.	
Usefulness	USE1	AI tools are beneficial for achieving my academic goals.	Davis (1989); Vankatesh and Davis (1996)
	USE2	I believe AI tools enhance my academic performance.	
	USE3	AI tools help me to complete my work more efficiently.	
	USE4	AI tools improve my productivity in academic tasks.	
Intention to Use	INT1	I believe AI tools will become an essential part of my learning process.	Davis (1989); Vankatesh and Davis (1996)
	INT2	If available, I will always choose to use AI tools.	
	INT3	I will recommend AI tools to other students for their learning.	
	INT4	I intend to continue using AI tools for my future learning.	

**Table 3:** Age by Gender.

	Age	
	Male	Female
Valid	97	88
Missing	0	0
Percentage	52%	48%
Mean Age	21.165	20.886
Std. Deviation	1.412	1.377
Minimum	19.000	20.000
Maximum	28.000	29.000

**Table 4:** Descriptive Statistics.

N = 185	Characteristics	Frequencies	Percentages
Computer Skills	No Skills	3	2%
	Beginners	73	39%
	Intermediate	95	51%
	Advanced	14	8%
AI Skills	No Skills	6	3%
	Beginners	95	51%
	Intermediate	76	41%
	Advanced	8	4%

**Table 5:** Related metrics, thresholds, and values.

Construct	Items	Factor Loadings > 0.7	Cronbach’s Alpha (CA) > 0.7	Composite Reliability (CR) > 0.7	Average Variance Extracted (AVE) > 0.5
Motivation	MOT1	0.794	0.812	0.877	0.641
	MOT2	0.864			
	MOT3	0.799			
	MOT4	0.741			
Self-efficacy	EFC2	0.912	0.779	0.901	0.819
	EFC3	0.899			
Enjoyment	ENJ1	0.837	0.891	0.924	0.754
	ENJ2	0.902			
	ENJ3	0.879			
	ENJ4	0.853			
Perceived Usefulness	USE1	0.889	0.916	0.941	0.799
	USE2	0.903			
	USE3	0.898			
	USE4	0.887			
Intention to use AI	INT1	0.881	0.928	0.949	0.823
	INT2	0.930			
	INT3	0.892			
	INT4	0.925			

**Table 6:** Discriminant validity using the Fornell–Larcker criterion.

	EFC	ENJ	INT	MOT	USE
EFC	**0.905**				
ENJ	0.534	**0.868**			
INT	0.405	0.673	**0.907**		
MOT	0.594	0.648	0.643	**0.801**	
USE	0.574	0.694	0.738	0.737	**0.894**

**Table 7:** Heterotrait–monotrait ratio (HTMT).

	EFC	ENJ	INT	MOT	USE
EFC					
ENJ	0.636				
INT	0.480	0.741			
MOT	0.746	0.755	0.741		
USE	0.680	0.764	0.798	0.852	

**Table 8:** Direct and mediating effects with hypotheses validations.

Hypotheses	Direct Path	Beta Value (β)	t-Value	*p* Value	Effect Size (F^2^)	Hypothesis Validation
H1	EFC -> MOT	0.594	8.914	0.000	0.546	Supported
H2	EFC -> ENJ	0.230	2.697	0.007	0.063	Supported
H3	MOT -> ENJ	0.512	7.067	0.000	0.311	Supported
H4	MOT -> USE	0.496	7.371	0.000	0.379	Supported
H5	ENJ -> USE	0.372	5.376	0.000	0.214	Supported
H6	USE -> INT	0.738	15.365	0.000	1.194	Supported

**Table 9:** Mediating effects with hypotheses validations.

Hypotheses	Indirect Path	Beta Value (β)	t-Value	*p* Value	95% BCa CI	Hypothesis Validation
H7	EFC -> MOT -> USE	0.295	5.389	0.000	[0.196, 0.414]	Supported
H8	EFC -> ENJ -> USE	0.086	2.235	0.025	[0.027, 0.182]	Supported
H9	EFC -> MOT -> ENJ -> USE	0.113	3.700	0.000	[0.066, 0.188]	Supported
Total Indirect Effect		0.494	-	-	-	

Note. Bootstrapping with 5000 subsamples; BCa = bias-corrected and accelerated confidence interval.

**Table 10:** Model fit.

	R^2^	Q^2^	Standardized Root Mean Square Residual (SRMR) < 0.08
**ENJ**	0.455	0.270	0.075
**INT**	0.544	0.151	
**MOT**	0.353	0.337	
**USE**	0.624	0.315	

## Data Availability

The datasets generated and analyzed in this study are not publicly available due to privacy, confidentiality, and related restrictions. However, they may be made available upon reasonable request to the corresponding author.
